# Book Reviews

**Published:** 1893-04

**Authors:** 


### *BOOK REVIEWS.*

Hand-Book of Massage. By Emil Kleen, Ph. D., Practicing Physician, Carlsbad, Bohemia. Authorized translation from the Swedish by Edward M. Hartwell, M. D., Director of Physical Training in the Public Schools of Boston. $2.75. P. Blakiston, Son & Co., Philadelphia.

The author states that he is no "Massage Specialist." He regards massage only as one "remedial measure, among many, that is capable of being frequently employed, but which is seldom to be resorted to by itself alone."

The first chapter gives the history and present standing of Massage. Others describe the technique (illustrated), the physiological and general therapeutic effects, contraindications, and its application in affections of the muscles, peripheral nerves, joint diseases, diseases of abdominal organs, genito-urinary organs, central nervous system, etc.

While Dr. Kleen's statements and claims for massage are conservative and free from the extravagance of the professional masseur, he does recognize in it a "procedure which is rightly coming to occupy a more and more prominent place in modern therapy."

There has been a need for a book describing the actual standing of massage from a scientific and clinical standpoint. This need, we think, is now supplied. The fact that it has received the high indorsement (in the introduction) of Dr. Weir Mitchell of Philadelphia, and Dr. Gustav Schutz of Berlin, is sufficient recommendation.

---

Treatise on Nervous and Mental Diseases, for Students and Practitioners of Medicine. By Landon Carter Gray, M. D., Professor of Nervous and Mental Diseases in the New York Polyclinic, etc. Published by Lea Brothers & Co., Philadelphia, 1893.

Dr. Gray has given to the world a book worthy of himself and his subjects. The only regret is that he has not been as exhaustive in treating mental diseases as he has nervous. The first part of this volume deals with the diseases of the nervous system. All the subjects are considered in a forcible, clear and correct manner. He has evidently been sincere in his effort, and has successfully considered all functional and organic troubles relating to the nervous system. No chapter is better than another, for all are good.

His handling of mental diseases is rather after the order of a synopsis, for though, so far as he goes, he does well, yet the subject of psychiatry is too vast to be dismissed with as few words as Dr. Gray gives thereto. The author is up to date in all his classifications, pathology and treatment. His diagnostic points are well drawn and reasonable. His treatment admirable. We only hope to hear from him more fully on psychiatry, for we doubt not he will give us a treatise on this subject as complete as he has on the nervous diseases. H. H.

---

Modern Homeopathy; Its Absurdities and Inconsistencies. By W. W. Browning, A. B., M. D., Brooklyn, N. Y. Published by Wm. J. Dornan, Philadelphia.

It will be remembered that last year Dr. Gould, of Philadelphia, offered a prize of one hundred dollars for the best essay on Homeopathy. To this pamphlet of thirty-two pages was awarded the prize. It is the best presentation of the absurdities of Homeopathy that we have ever read. The author does not resort to ridicule—that would be no argument—but in a dignified manner exposes the unreasonableness of the Homeopathic faith from the words of its own disciples. He sets down naught in malice; neither does he extenuate. It is an excellent description of the absurdities and inconsistencies of the Homeopathic heresy. Physicians and the public also should inform themselves better as to the faith of this school. It is now dying out, but it will die slowly, like any other delusion. Copies of the pamphlet may be ordered of Dr. Geo. M. Gould, 119 S. Seventeenth St., Philadelphia, at the rate of seventy-five cents per dozen.

A HAND-BOOK OF THE DISEASES OF THE EYE, AND THEIR TREATMENT. By Henry R. Swanzy, A. M., M. B., F. R. C. S. I. Surgeon to the National Eye and Ear Infirmary, and Ophthalmic Surgeon to the Adelaide Hospital, Dublin; Examiner in Ophthalmic Surgery in the University of Dublin. Fourth Edition. With Illustrations. P. Blakiston, Son & Co., Philadelphia.

This book continues to find favor with the profession, as an evidence of which the third edition was published in October, 1890, and the fourth was published in less than two years afterwards to meet the increasing demand for the work. The author possesses to a remarkable degree that happy faculty of presenting a technical and voluminous subject in a concise, bright and entertaining manner; and, to use his own words, "saying not all he might but all he ought; giving a succinct and practical account of his subject in its most modern aspect, without weighing his pages with excessive detail and prolonged discussion." It is a work of genuine merit and will maintain its position as one of the standards.

---

THE PHYSICIAN; HIS RELATION TO THE LAW. By H. G. Blaine, A. M., M. D., Late Professor of Diseases of the Nervous System in the Toledo Medical College, Member of the Northwestern Ohio Medical Association. Blaine Bros., Toledo, Ohio.

The author says, "the object of this little pamphlet is to give in a condensed form the relation of the physician to the law, and to give him a more extended knowledge of the rules governing the collection of his fees." This object has been very well carried out. The book contains many suggestions and legal points of every-day interest and value to the physician. Such subjects as malpractice, confidential relation to patient, authority in emergencies, legal rules governing the collection of fees, responsibility for payment, as a witness in court, etc., are well discussed in their legal bearings; the whole making up a very useful and practical little work for the physician.

HAND-BOOK OF INSANITY for Practitioners and Students. By Dr. Theodore Kirschoff, Physician to the Schleswig Insane Asylum, and private Docent at the University of Kiel. Published by William Wood & Company, New York, 1893.

This work deserves sale. It is gotten up in good form, the plates are good, and the data correct. There is nothing of an original character contained therein, but the views and expressions of different writers have their worth, and in view of this fact none could go wrong either in purchasing or reading the work. We might with justice say, that though not original, the author will reach a class of readers who would never study the more exhaustive works on insanity, and in this way do a vast deal of good, for no one branch of medicine is so little read or studied as psychiatry, and no one branch is of more importance either to the medical or social world. H. H.

---

A MANUAL OF CLINICAL OPHTHALMOLOGY. By Howard F. Hansell, M. D., Lecturer on Ophthalmology, and James H. Bell, M. D., Member Ophthalmological Staff Jefferson Medical College. 120 Illustrations. P. Blakiston, Son & Co., Philadelphia.

The authors state in the preface that it has been their "purpose to place before the undergraduate and general practitioner of medicine a brief review of the anatomy, physiology, refraction and common diseases of the eye. No attempt has been made to treat the subjects exhaustively, but an object of directness and practicability of clinical teaching and practice." The diagrams are especially good, and clearness of expression has not been sacrificed for the sake of brevity.

---

### PRELIMINARY PROGRAMME OF THE FORTY-FOURTH ANNUAL SESSION OF THE MEDICAL ASSOCIATION OF GEORGIA TO BE HELD IN AMERICUS, GEORGIA, APRIL 19, 20 AND 21, 1893.

---

### PAPERS TO BE READ.

---

(*Partial List.*)

The President's Annual Address, A. A. Smith, M. D., Hawkinsville; Orator's Address—Woman's Relations to the Practice of Medicine, Frank Ridley, M. D., LaGrange; Diffuse Traumatic Aneurism of Anterior Tibial Artery—Ligation of Femoral, F. R. Calhoun, M. D., Cartersville; Multiple Neuritis "Alcoholic," Mark H. O'Daniel, M. D., Macon; Salpingitis: Pathology and Treatment, R. R. Kime, M. D., Atlanta; Puerperal Eclampsia, with Special Reference to its Cause and Treatment, A. C. Davidson, M. D., Sharon; Asphyxia Neonytorum, R. J. Nunn, M. D., Savannah; Stab of the Stomach—The Organ Protruding through Abdominal Wall—Laparotomy—Recovery, J. W. Griggs, M. D., West Point; Operation for Fistula in Ano by Ligation, John J. Hill, M. D., Washington; The Contagiousness of Consumption, J. G. Hopkins, M. D., Thomasville; The Disappearance of the Menopause, J. C. Avary, M. D., Atlanta; Headache Versus Glaucoma, W. T. Bullard, M. D., Columbus; "Shot Gun" Prescriptions, C. C. Hart, M. D., Cross Keys; A Review of "Dr. Senn's Views on Elastic Constrictions," W. H. Elliot, M. D., Savannah; The Practice of Medicine in Georgia, A. C. Blain, M. D., Macon; Hernia, W. F. Westmoreland, M. D., Atlanta; Antipyretics, Translated from the German, S. B. Poland, M. D., Griswoldville; Science in Medicine and Surgery, J. McFadden Gaston, M. D., Atlanta; Ophthalmia of the New Born, S. Latimer Phillips, M. D., Savannah; Periproctitis with an Abscess, and Report of a Case, M. L. Currie, M. D., Mt. Vernon; The Necessity for a Medical Examining Board in Georgia, L. B. Grandy, M. D., Atlanta; Impure and Pure Mineral Waters, T. S. Hopkins, M. D., Thomasville; State and Municipal Hygiene, J. C. Avary, M. D., Atlanta; Rare Case in Obstetric Practice, O. H. Buford, M. D., Cartersville; Sterility in the Male, C. Evans Johnson, Atlanta; Conditions Indicating Abdominal Operations and Report of Cases, Frank M. Ridley, M. D., LaGrange; Pneumonia, O. T. Kenyon, M. D., Weston; The Function and Nutritive Value of Foods, Louis H. Jones, Atlanta; Stone in the Bladder, with Report of Cases, F. W. McRae, M. D., Atlanta; *Non*-Surgical Treatment of Typhlitis, E. H. Richardson, M. D., Atlanta; Drainage in Pelvic Surgery, Geo. H. Noble, M. D., Atlanta; Specialism in Medicine, A. S. Hawes, M. D., Atlanta; Partial Tenotomy a Radical Cure for Heterophoralgia, C. H. Peete, M. D., Macon; Pathology of Gynecic Neurosis, Ross P. Cox, M. D., Rome; Persistent Remittent or so-called Typho-Malarial Fever, with Report of Cases, W. P. Williams, M. D., Blackshear; Some Remarks on Aseptic Surgery, with Exhibition of Sterilizing Methods, T. M. McIntosh, M. D., Thomasville; Mechanical Treatment of Some Skin Anomalies, M. B. Hutchins, M. D., Atlanta; Three Women who Refused Laparotomy, H. McHatton, M. D., Macon.

DAN H. HOWELL, M. D., *Secretary.*  A. A. SMITH, M. D., *President.*

